# Chemopreventive and chemotherapeutic effects of dietary supplementation of vitamin D on cholangiocarcinoma in a Chemical-Induced animal model

**DOI:** 10.18632/oncotarget.2000

**Published:** 2014-05-22

**Authors:** Kun-Chun Chiang, Chun-Nan Yeh, Kun-Ju Lin, Li-Jen Su, Tzu-Chen Yen, Jong-Hwei S. Pang, Atsushi Kittaka, Chi-Chin Sun, Miin-Fu Chen, Yi-Yin Jan, Tai C. Chen, Horng-Heng Juang, Ta-Sen Yeh

**Affiliations:** ^1^ General Surgery Department, Chang Gung Memorial Hospital, Keelung, Taiwan, ROC; ^2^ General Surgery Department, Chang Gung Memorial Hospital, Linkoul, Taoyuan, Taiwan, ROC; ^3^ Nuclear medicine department, Chang Gung Memorial Hospital, Linkoul, Taoyuan, Taiwan, ROC; ^4^ Institute of Systems Biology and Bioinformatics, National Central University, Jhongli City, Taoyuan, Taiwan, ROC; ^5^ Graduate Institute of Clinical Medical Sciences, College of Medicine, Chang Gung University, Kwei-Shan, Taoyuan, Taiwan, ROC; ^6^ Faculty of Pharmaceutical Sciences, Teikyo University, 2-11-1Kaga, Itabashi, Tokyo, Japan; ^7^ Department of Ophthalmology, Chang Gung Memorial Hospital, Keelung, Taiwan, ROC; ^8^ Boston University School of Medicine, Boston, MA, USA; ^9^ Department of Anatomy, College of Medicine, Chang Gung University, Kwei-Shan, Taoyuan, Taiwan, R.O.C

**Keywords:** cholangiocarcinoma, vitamin D, chemoprevention, LCN2, NGAL

## Abstract

Intrahepatic cholangiocarcinoma (ICC) is an aggressive cancer. Vitamin D supplementation is getting popular due to its anti-tumor functions after conversion to its active form, 1α,25(OH)_2_D. Here, we show that dietary supplementation with 6 IU/g of vitamin D greatly suppressed ICC initiation and progression without apparent toxicity in a chemically induced rat model. Microarray analysis of rat ICC tissues showed vitamin D supplementation modulated the expressions of several unique genes, including lipocalin 2 (Lcn2), confirmed by RT-qPCR and immunohistochemical (IHC) staining. Further, 53 of 80 human ICC specimens (66%) exhibited high LCN2 expression and LCN2 knockdown in SNU308 cells decreased cell growth and migration, suggesting LCN2 be an oncogene in human ICC. As human ICC SNU1079 cells were treated by 1α,25(OH)_2_D_3_, LCN2 expression and cell proliferation were attenuated. The downregulation of LCN2 expression was blunted when vitamin D receptor (VDR) was knocked down, implicating that the *in vivo* Lcn2 downregulation is a direct consequence of vitamin D supplementation

Our results support the prevailing concept that vitamin D status is negatively associated with cancer incidence and mortality and suggest LCN2 may be a potential target against ICC. Further studies of application of vitamin D or its analogs against ICC are warranted.

## INTRODUCTION

Cholangiocarcinoma (CCA), the second most common malignancy in the liver after hepatocellular carcinoma, originates from the epithelial lining of biliary tract [1-3] with increasing incidence and mortality recently [1-5]. Intrahepatic cholangiocarcinoma (ICC) originates from the small bile ducts within the liver. The survival of ICC is very poor in general and only surgical resection can provide a cure in the case of early stage of disease [6-8], which is rare due to the lack of early diagnostic methods. For unresectable ICC [9], the prognosis is dismal with the average survival of less than one year [10], attributable to the resistance to traditional chemotherapy and radiotherapy, Recently, target therapy has been shown to have potential being a promising strategy against CCA[11, 12]. Collectively, clinicians face a stalemate to deal with patients with advanced ICC. New therapeutic strategies to deal with ICC are indeed urgently needed.

Vitamin D, originally known to modulate calcium absorption and bone metabolism for nearly a century, is now shown to have potent antiproliferation, antiangiogenesis, anti-inflammation, pro-apoptosis, pro-differentiation, and immune-regulation in many cells through a cell- and tissue-specific manner [13-18]. Moreover, evidence associating vitamin D deficiency, defined as serum 25(OH)D concentration less than 20 ng/ml in human, with the increased incidence of prostate, colon and breast cancers in a number of ecological and epidemiological studies has been well documented [19-22], although some studies have questioned their association[23]. Vitamin D exerts its transcriptional regulation through binding to vitamin D receptor (VDR), which forms a hetorodimer with retinoid X receptor (RXR), and binds to vitamin D response element (VDRE) located in the promoter region of vitamin D responsive genes to transactive gene expression [24, 25]. Since VDR is present in almost all tissues studied, the application of vitamin D as a new generation of tumor preventive and therapeutic agents is plausible.

Regarding CCA, 1α,25(OH)_2_D, the active form of vitamin D, has been shown to inhibit CCA cell growth *in vitro* [26], and dysregulation of the local conversion of 25-hydroxyvitamin D to 1,25-dihydroxyvitamin D, the hormonal form of vitamin D, may lead to enhanced CCA [27]. Moreover, VDR expression has been demonstrated in cultured CCA cell lines and CCA tissues, in which high levels of expression were associated with good prognosis of CCA [26]. These findings suggest that vitamin D deserves further investigation against CCA. So far, no study concerning the application of dietary vitamin D supplementation against CCA *in vivo* has been published.

Previously we have established a thioacetamide (TAA)-induced rat ICC model [28], in which the tumors can be induced after feeding rats with TAA-containing water for 20 weeks. The model successfully recapitulates human ICC progression histologically. Furthermore, the tumor growth can be easily evaluated by a micro PET for small animals [29]. In this report, we investigated the effect of vitamin D supplementation with 2 IU/g and 6 IU/g vitamin D_3_ (cholecalciferol) on ICC via the TAA-induced ICC animal model. The potential underlying mechanisms were evaluated through cDNA microarray analysis and the possibly responsible gene was further verified in human ICC specimen and human ICC cell lines.

## RESULTS

### Measurement of body weight, serum calcium and 25(OH)D levels of rats

**Figure 1:**
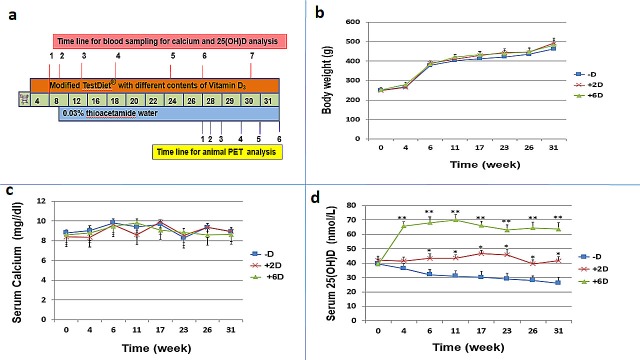
Animal study protocol and Measurement of body weight, serum calcium, and serum 25(OH)D concentration of rats during the study period a. Animal study protocols and measurements of biomarkers b.Animals (N=7 per group) were fed with a diet containing either no vitamin D (-D), 2 IU/g vitamin D_3_ (+2D), or 6 IU/g vitamin D_3_ (+6D). Rats in three groups all gained body weight increase stably and no significant difference was observed among the 3 groups. c. No significant difference was observed among the 3 groups regarding serum calcium concentration. d. The serum 25(OH)D level varied significantly in each group in accordance with the vitamin D supplementation amount. Each points is the mean±SD of 7 animals. *p<0.05, **p<0.01.

As described in the diagram shown in Fig. 1a, animals were divided into three groups (N=7 per group) and fed with a diet supplemented with no vitamin D_3_, 2 IU/g of vitamin D_3_, or 6 IU/g of vitamin D_3_ and kept from exposure to ultraviolet radiation b (UVB) to prevent endogenous vitamin D synthesis. Drinking water was replaced with water containing 0.03% TAA beginning in week 8.

The measurements of body weight, serum calcium and serum 25(OH)D were performed in week 1, 4, 6, 11, 17, 23, 26, and 31 for diets added with no vitamin D_3_ (-D), 2 IU/g (+2D) and 6 IU/g (+6D) vitamin D_3_ groups. No significant difference in body weight was observed among the three groups (Fig. 1b). All animals gained weight quickly until they drank TAA-containing water, and the increase of their body weight became more gradual afterward (Fig. 1b). Similarly, serum calcium stayed relatively unchanged and no significant difference was observed among the three groups during the entire study period (Fig. 1c). However, the serum 25(OH)D levels varied depending on the vitamin D_3_ amount in the diets (Fig. 1d). It increased from 40±3 to 60-70 nmol/L by week 4 and stayed in this range in the +6D group, whereas it remained relatively constant at about 43±3 nmol/L in the +2D group, and gradually declined from 40±3 to 26±4 nmol/L in the -D group.

### Evaluation of tumor initiation and progression by position emission tomography (PET) scanning

**Figure 2:**
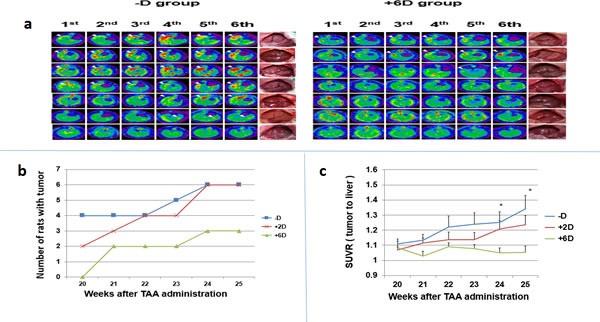
Evaluation of tumor occurrence and progression by micro animal PET a.The 6 times of PET images taken from rats in -D (left panel) and +6D (right panel) groups. The white arrows indicated the tumor with highest FDG uptake. The gross view of liver of each rat is shown in the right side. b.The number of rats bearing PET-detectable tumors. c. Due to the limitation of micro PET to detect tumor with size < 2 mm and the indistinguishable border between normal tissues and invasive CCA, the standard tracer uptake value ratio (SUVR, tumor to liver)was obtained to represent tumor growth.The SUVR, tumor to liver, was calculated from micro PET scan. Each points is the mean±SD of 7 animals. *p<0.05, **p<0.01

Previously, we have shown that ICC can be induced in rats after drinking water containing 0.03% TAA for 20 weeks with a highly successful rate [28]. Weekly PET scan began in the 20^th^ week after the initiation of TAA treatment. Figure 2a compares 2-deoxy-2-[F-18]fluro-D-glucose (FDG) images taken consecutively (1^st^ to 6^th^ scan) for 6 weeks between the -D and +6D groups. The scanning results from the 3 groups are summarized in Figure 2b. In the 1^st^ scan, no rat bearing tumors was detected in the +6D group, while 2 and 4 rats were found bearing tumors in the +2D and -D groups, respectively. In the 5^th^ and 6^th^ scans, the +6D group had 3 rats with tumors, and +2D and -D groups each had 6 animals with tumors. Due to the limitation of micro PET to detect tumor with size < 2 mm and the indistinguishable border between normal tissues and invasive CCA, the standard tracer uptake value ratio (SUVR, tumor to liver) was obtained to represent tumor growth [29]. As shown in Figure 2c, the -D group had the highest SUVR in the 1^st^ scan which increased gradually in the following scans during the study. The +2D group also showed increased SUVR but with a lower magnitude than the -D group. However, the SUVR for the +6D group stayed relatively unchanged and was significantly lower than the -D group in the 5^th^ and 6^th^ scans.

### Genome-wide gene expression profile analysis for rat ICC tissues from –D, +2D and +6D animals

**Figure 3:**
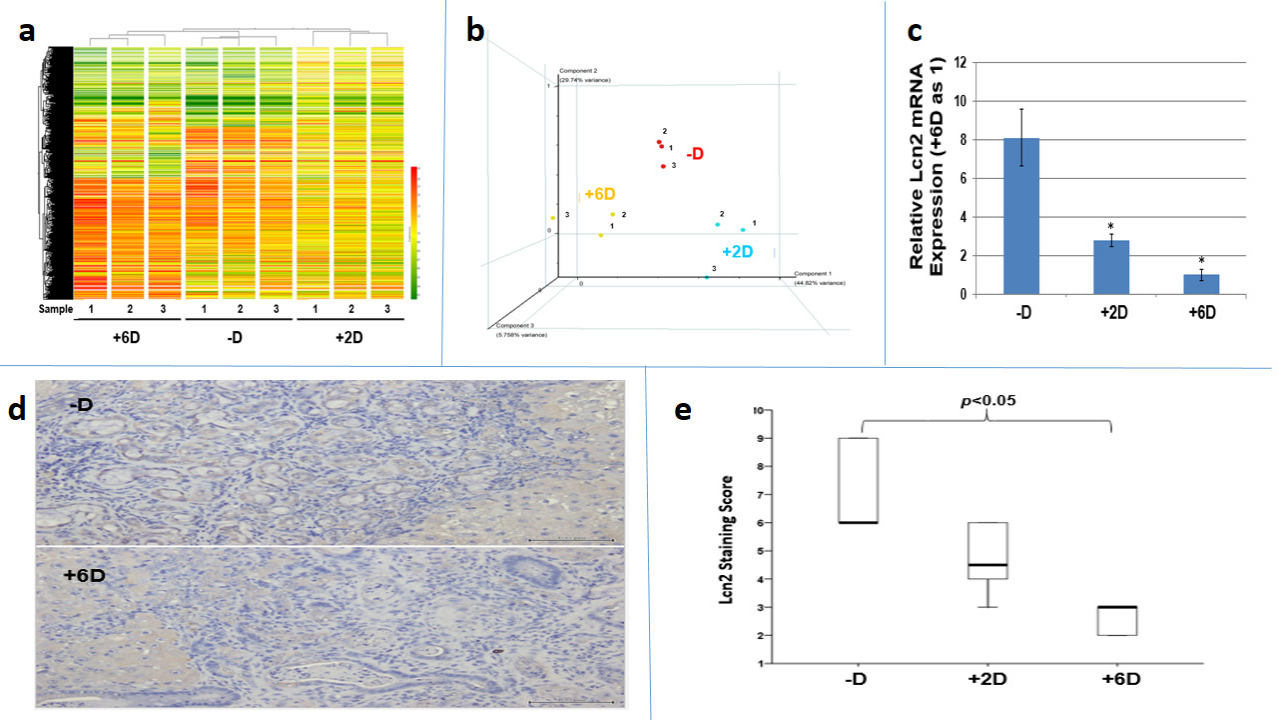
Analyses of tumor tissues obtained from -D, +2D and +6D groups of rats by microarray, qRT/PCR and immunohistochemistry (IHC) staining a. Molecular portrait of gene expression profile in rat ICC (-D, +2 IU and +6D groups). Hierarchical clustering illustrates 1,633 gene expression patterns. The results are shown in a diagram format, in which rows represent individual transcripts and columns represent data from 3 different animals in each group. The color in each cell reflected the expression level of the corresponding sample, relative to its mean expression level. The result indicated each group has indistinct gene expression profiles and the similar gene expression profile is observed within the group. b Principal components analysis (PCA) of vitamin D responsive gene expression profiles. The gene expression in each group was analyzed by PCA method. The individual point from -D, +2D and +6D group was marked in red, blue, and orange, respectively. c. *Lcn2* mRNA expression in tumor tissues. Comparison of *Lcn2* mRNA expression by RT-qPCR among tumors from -D, +2D and +6 D animals. *p<0.05 d. *LCN2* expression in tumor tissues. Comparison of *Lcn2* expression by IHC staining between the tumors from -D and +6D animals. e. Quantitative analysis of tumor tissue by IHC staining. Box plots analysis was used to compare the *LCN2* IHC staining among tumors from -D, +2D and +6D animals. The *LCN2* expression in –D and +6D group is significantly different.

The potential mechanism involved in the vitamin D-dependent anti-ICC actions was investigated by microarray analyses using Rat OneArray^®^ v1 to compare the gene expression profiles in tumors from these three groups of rats. As shown in Fig. 3a and 3b, vitamin D supplementation did induce indistinct gene expression profiles *in vivo*. (The methods and data analysis for microarray were described in the supplementary materials section). Among the downregulated genes, *Lcn2* was found to be the most suppressed by vitamin D supplementation with a 38% and 50% reduction in expression, respectively, in the tumors from +2D and +6D rats compared to the -D rats (Table 1a and Supplemental Fig.S-1).

**Table 1a:** Genes affected by vitamin D supplementation

Probe set ID	Gene name	Vitamin D supplementation		
		-D	+2D	+6D
Up regulated				
PH_rn_0012953	Arsj	0.25	0.77	1.35
PH_rn_0020767	Ces1d	0.41	0.81	0.81
PH_rn_0003591	Havcr1	0.63	1.08	1.15
PH_rn_0001350	Ccdc144b	0.27	0.45	0.62
PH_rn_0003985	Gucy1b2	0.34	0.80	1.31
PH_rn_0003024	Aldh1l1	0.52	0.82	1.30
PH_rn_0022686	NA	0.80	1.59	2.72
PH_rn_0014388	LOC287167	0.23	1.15	1.42
PH_rn_0014705	Tnfsf4	0.63	1.10	1.29
PH_rn_0009406	Pipox	0.53	0.87	1.58
PH_rn_0010923	Cyp7a1	0.35	0.82	1.64
PH_rn_0014336	LOC305806	0.58	1.06	2.26
PH_rn_0004153	Trim54	0.59	0.98	1.31
PH_rn_0003819	Nr1i3	0.63	1.21	2.27
PH_rn_0016772	Rergl	0.67	1.16	1.80
PH_rn_0022202	LOC100360253|LOC100364984	0.83	1.47	1.88
PH_rn_0002165	Cyp2c11	0.17	0.95	1.66
PH_rn_0008613	Sdr16c6	0.52	0.89	1.98
PH_rn_0018922	Gkn3	0.95	1.34	2.01
PH_rn_0022007	Wisp3	0.71	1.31	1.93
PH_rn_0001170	LOC100360120	0.26	0.46	0.62
Down regulated				
PH_rn_0020605	Abcc1	1.61	1.06	0.77
PH_rn_0016277	LOC686596	0.60	0.71	0.57
PH_rn_0001393	Cd207	1.66	1.13	0.44
PH_rn_0001576	Stra8	0.47	0.38	0.20
PH_rn_0007276	Spink4	2.09	1.22	1.07
PH_rn_0003053	Pdpk1	1.63	0.97	0.63
PH_rn_0020801	RGD1565486	1.55	0.84	0.63
PH_rn_0010729	Ereg	1.31	1.06	0.82
PH_rn_0010578	Lcn2	1.72	0.87	0.33
PH_rn_0002779	Slc26a3	1.90	1.05	1.02
PH_rn_0008583	Fkbp5	1.72	0.84	0.66
PH_rn_0015419	Taar9	0.95	0.42	0.29
PH_rn_0002499	LOC681122	0.34	0.33	0.20
PH_rn_0008827	Cnnm1	0.34	0.20	0.16
PH_rn_0004742	B3gat2	0.21	0.18	0.15
PH_rn_0009834	Cts7	0.87	0.51	0.20

**Table 1b:** Pathways affected by vitamin D supplementation

Signal Pathway		odds radio (loge)	p value	Adjusted p value
Up regulated				
	Metabolism of xenobiotics by P450	2.58	<0.0001	<0.0001
	Drug metabolism - P450	2.87	<0.0001	<0.0001
	Metabolic pathways	1.20	<0.0001	<0.0001
	Primary bile acid biosynthesis	3.39	<0.0001	<0.0001
	PPAR signaling pathway	2.53	<0.0001	<0.0001
	Glycosphingolipid biosynthesis	2.85	0.0017	0.0192
	Retinol metabolism	2.97	<0.0001	<0.0001
	Linoleic acid metabolism	3.33	<0.0001	<0.0001
	Drug metabolism	2.36	<0.0001	0.0003
	Arachidonic acid metabolism	2.40	<0.0001	<0.0001
Down regulated				
	Retinol metabolism	1.90	0.0005	0.0265
	ABC transporters	2.19	0.0005	0.0265
	Biosynthesis of unsaturated fatty acids	2.21	0.0017	0.0449
	Tyrosine metabolism	2.38	0.0002	0.0265
	Focal adhesion	1.33	0.0011	0.0337
	Taste transduction	1.83	0.0020	0.0470
	mTOR signaling pathway	2.15	0.0006	0.0265
	Glycosaminoglycan biosynthesis	3.02	0.0010	0.0337

### Verification of *Lcn2* expression in rat ICC tissues and *LCN2* expression in human ICC tissues

The downregulation of *Lcn2* expression by vitamin D supplementation *in vivo* as indicated by microarray were confirmed by RT-qPCR (Fig. 3c) and IHC staining (Fig. 3d and 3e), which showed that both of *Lcn2* mRNA and protein expressions were significantly reduced in ICC tumors from +6D group compared to those from -D group. We further examined 80 human ICC tissues and found that 53 (66%) specimens exhibited high LCN2 expression compared to the non-tumorous bile ducts (Supplemental Fig S-2).

### Evaluation of the expression of VDR, and antiproliferative effect of 1α, 25(OH)_2_D_3_ on Human ICC cell line, SNU 1079

**Figure 4:**
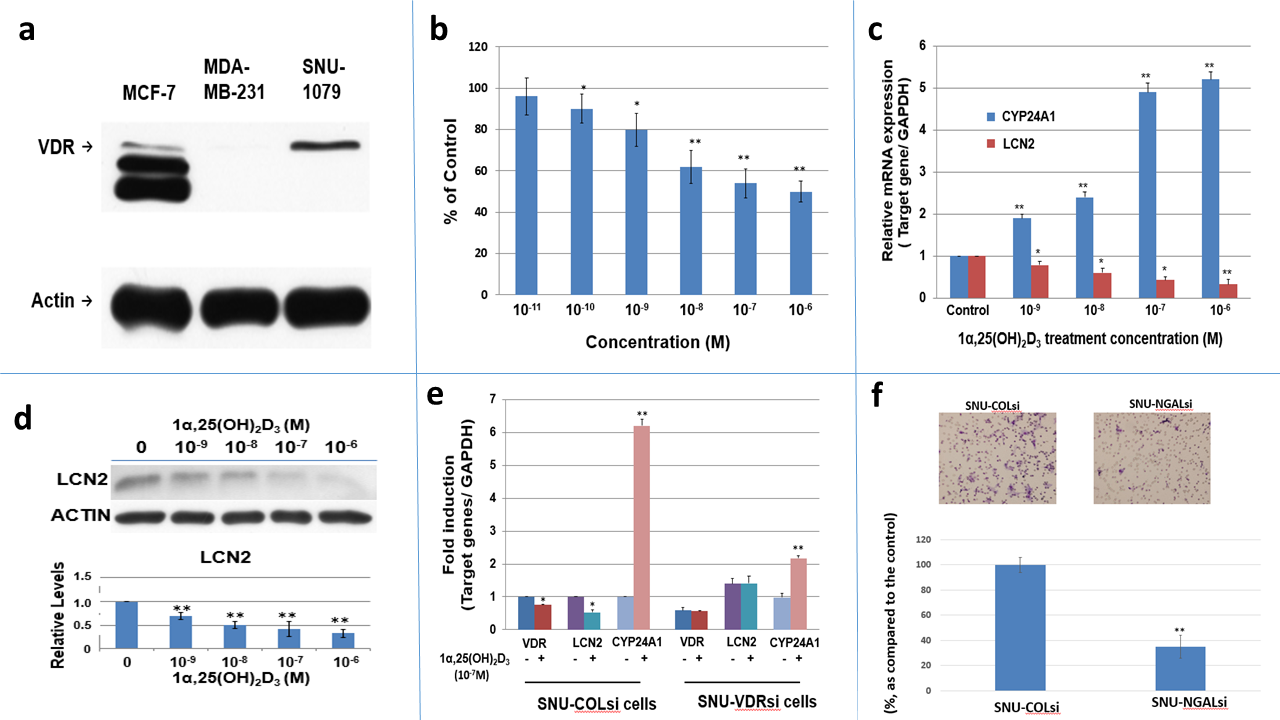
The expression of VDR, LCN2 mRNA and protein in SNU1079 human ICC cells and evaluation of 1α,25(OH)D effect on SNU1079 cells a. Western blot of VDR in SNU1079 cells. VDR expression in SNU1079 cells. MCF-7 cells and MDA-MB-231 cells were used as a positive and negative control, respectively. b. Dose-dependent inhibition of SNU1079 cell proliferation by 1α,25(OH)_2_D_3_.1α,25(OH)_2_D_3_, from 10^−6^M to 10^−10^M, repressed SNU1079 cell proliferation dose dependently. Each point represents the mean ± SD of 3 wells from a single experiment. *p<0.05, **p<0.01 c. A dose-dependent downregulation of ***LCN2*** mRNA expression by 1α,25(OH)_2_D_3_.Treatment of 10^−6^ to 10^−9^M1α,25(OH)_2_D_3_ repressed *LCN2* mRNA expression in SNU1079 cells dose dependently. CYP24A1 was used as the positive control. *p<0.05, **p<0.01 d. A dose-dependent downregulation of *LCN2* expression by 1α,25(OH)_2_ D_3_.Treatment of 10^−6^ to 10^−9^ M1α,25(OH)_2_D_3_ repressed LCN2 expression in SNU1079 cells dose dependently. (60 μg protein was loaded in each well and actin was applied as an internal control) Data are presented as the fold change (mean ± SE of three independent experiments) of the protein levels in relation to the control group. *p<0.05, **p<0.01 e. The consequence of VDR knockdown on the 1α,25(OH)_2_ D_3_ -induced *LCN2* mRNA expression. SNU-1079 cells were transduced with control non-target shRNAlentiviral particles (SNU-COLsi) or VDR shRNAlentiviral particles (SNU-VDRsi) for 96 hours and then were treated with 10^−7^ M of 1α,25(OH)_2_D_3_or control ethanol vehicle for another 24 hours. The LCN2, CYP24A1, and VDR mRNA levels were determined by RT-qPCR. CYP24A1 was used as a positive control. The mRNA level of control group was set as 1. Data are presented as the fold change (mean ± SE of three independent experiments) of the mRNA levels in relation to the control group. *p<0.05, **p<0.01 f.The consequence of *LCN2* knockdown on SNU308 cell migration Cell migration of SNU-COLsi cells (mock knockdown SNU308 cells) and SNU-NGALsi cells (*LCN2* knockdown SNU308 cells) was measured by using non-matrigel-coated membrane as described in the Materials and Methods. The number of migrating cells was digitally photographed and counted under the microscope (IX71, Olympus, Tokyo, Japan). Experiments were performed in triplicate and repeated at least three times. (* P<0.05, ** P<0.01)

We utilized a human ICC cell line, SNU1079, a VDR-expressing cell lines (Fig 4a), to investigate whether this cell line was responsive to the antiproliferative effect of 1α,25(OH)_2_D_3_. As shown in Figure 4b, 1α,25(OH)_2_D_3_ significantly inhibited SNU1079 cell growth in a dose-dependent manner at the concentrations from 10^−10^ to 10^−6^ M after 4 days of treatment.

### Evaluation of the effect of 1α, 25(OH)_2_D_3_ on *LCN2* expression in SNU1079 cells

We next investigated whether human *LCN2* expression was regulated by 1α,25(OH)_2_D_3_ and whether the effect was mediated through VDR in SNU1079 cells. Using RT-qPCR and western blot analyses, we found that 1α,25(OH)_2_D_3_ caused a dose-dependent downregulation of LCN2 mRNA and protein expression in SNU1079 cells with dramatic upregulation of CYP24 mRNA expression, the most inducible gene expression by 1α,25(OH)_2_D_3_ (Fig. 4c and 4d). Furthermore, we showed that VDR knockdown by shRNA blunted the 1α,25(OH)_2_D_3_-dependent *LCN2* mRNA downregulation (Fig. 4e). Given that the added FBS in cell culture medium contained some amount of 1α,25(OH)_2_D_3_, the higher *LCN2* expression in SNU-VDRsi cells than that of SNU-COLsi cells also indicated the downregulation of LCN2 by 1α,25(OH)_2_D_3_ is VDR-mediated (Fig 4e).Consequently, we concluded that *LCN2* expression is regulated by 1α,25(OH)_2_D_3_ and is mediated through VDR in SNU1079 cells.

### Evaluation *LCN2* effect on cell growth and migration of SNU308 cells

To further determine *LCN2* effect on human CCA cells, we knockdowned *LCN2* in another CCA cell line, SNU308, and obtained SNU-COLsi cells (mock knockdown SNU308 cells) and SNU-NGALsi cells (*LCN2* knockdwon SNU308 cells)(Figure S-3). The cell doubling time was calculated by two time points of cell number. The cell doubling time was increased to 30.45 hours from 23.6 hours and the migration ability is significantly repressed in SNU308 cells after *LCN2* knockdown (Fig 4f).

## DISCUSSION

CCA is a devastating disease with increasing incidence and mortality in recent years and with very few therapeutic options [3-5]. Since the active form of vitamin D, 1α,25(OH)_2_D_3_ or calcitriol, has been well demonstrated to be a pleiotropic hormone with a variety of anti-tumor actions [17, 30], combined with the fact that vitamin D deficiency has been associated with a number of cancers incidence [22], we thus investigated whether vitamin D supplementation could benefit the prevention of ICC initiation and progression under the bleak background of ICC treatment. Using microarray approach, a recent publication reported that vitamin D supplementation to normal subjects can affect many cancer related genes [31], supporting the epidemiological findings that vitamin D-deficiency may be associated with many forms of cancer [22], and also suggests the importance of adequate vitamin D nutrition in preventing cancers. Because of the concern about the UV-induced skin cancers, humans have not generated sufficient vitamin D from sunlight exposure and increasingly depend on the dietary supplementation. However, how much vitamin D do we really need to prevent cancers is still unknown [32].

In this study, we showed that the rat serum 25(OH)D level, the best index of vitamin D status, changed with time depending on the contents of vitamin D in their diets (Fig. 1d). As shown, supplementation with 6 IU/g vitamin D_3_ increased the serum 25(OH)D to a much higher level (about 50% increase), whereas there was a gradual decline to vitamin D-deficient state in the -D animals. There was no significant changes in serum 25(OH)D in the +2D group fed with 2 IU/g vitamin D_3_ diet. In spite of differences in serum 25(OH)D levels among the three groups, there was no significant difference in their body weight and serum calcium over the course of study (Fig. 1b and 1c). This observation, showing no adverse effects on body weight and serum calcium, indeed indicates that supplementation with 6 IU/g of vitamin D_3_ did not cause unwanted side-effects even though they had serum 25(OH)D higher than the normal range [33]. Furthermore, the lack of increase in serum calcium in 6 IU/g group demonstrates that any anti-ICC effects observed in this group are most likely a direct consequence of vitamin D supplementation, and not a secondary effect attributable to calcium [34, 35].

Our data, for the first time, clearly showed an anti-ICC initiation and progression (Fig. 2a, b and c) by supplementing animals with 6 IU/g of vitamin D_3_ which also raised their serum 25(OH)D level to between 60 and 70 nmol/L (Fig. 1d) in this TAA-induced rat ICC model. The tumor incidence and tumor progression in –D and +2D groups are similar (Fig 2b&c), whereas +6D group has significantly lower tumor incidence and progression as compared to –D group (Fig. 2a, b, &c). Since the vitamin D content in the regular rat chows which contain 2.2 IU/g vitamin D [33] is comparable to our special 2 IU/g diet, apparently, the normal level of vitamin D in the diet or normal circulating level [33] of 25(OH)D may not be sufficient for preventing ICC tumor initiation and progression (Fig. 2a, b, &c). This finding is consistent with a prevailing view that a higher level of dietary reference intakes (DRI) than what is needed for bone health [36] may be required for the prevention of cancers and other chronic diseases[22, 32, 37].

Functional genomic analyses will probably have multiple implications for candidate drug discovery against targeted genes. At the present time, 408 upregulated and 230 downregulated genes have been identified as potential vitamin D targets by the use of chromatin immune-precipitation DNA sequencing (ChiP-seq) analysis [38]. Thus, to further understand the underlying mechanisms whereby vitamin D exerts its chemopreventive and chemotherapeutic effects on ICC, cDNA microarray was conducted. By using the Rat OneArray^®^ which covers 24,358 well-substantiated rat transcripts, we performed the genome-wide mRNA microarray on ICC tumors from each of the three groups under study. The arrays demonstrate a unique expression profile pattern for the triplicate samples from each group (Fig. 3a & b), indicating different vitamin D supplementations did change the gene expression profile. After a series of bioinformatical analyses [39] (Please reference the supplemental material and method), we identified 21 and 16 genes which were significantly upregulated and downregulated, respectively, in response to vitamin D_3_ supplementation (Table 1a). Signal pathway analysis showed the vitamin D-upregulated pathways are mainly involved in fatty acid, bile acid, vitamin A and drug metabolic pathways, whereas the vitamin D-downregulated pathways include those responsible for mTOR signaling pathway, glycosaminoglycan biosynthesis, adhesion, tyrosine, vitamin A and fatty acid metabolic pathways (Table 1b).These genes or pathways might be used as targets for finding novel drug for cholangiocarcinoma treatment, and be used in clinical diagnosis in the future studies.

Among the downregulated genes found in our microarray analysis, *LCN2* was found to be the most suppressed by vitamin D supplementation. The results of IHC staining and RT-qPCR of TAA-induced rat ICC tissues confirmed the microarray data (Fig 3c,d,&e). *LCN2*, a member of lipocalin family that transports small, hydrophobic ligands, is a 25-kDa glycoprotein originally isolated from human neutrophils, therefore, it is commonly known as neutrophil gelatinase-associated lipocalin (NGAL) [40]. The protein is also known as neu-related lipocalin, aka SIP24, oncogene 24p3, uterocalin and siderocalin [40]. It is expressed in several normal tissues where its roles are mainly to protect against bacterial infection and oxidative stress [41]. *LCN2* expression is dysregulated in some benign and malignant diseases, and has been shown to play multifaceted roles in cancer in a cell lineage specific manner [42]. In malignant cells, its functions may include inhibiting apoptosis, promoting invasion and angiogenesis, and increasing proliferation and metastasis. The levels of *LCN2* have also been shown to be elevated by several orders of magnitude during injury, infection and malignancy, indicating a role it may play in tumor development [41, 42].

Regarding cholangiocarcinoma (CCA), a study reported that *LCN2* expression was detected in the CCA cell line, HuCCA-1, but not in 4 hepatocellular carcinoma cell lines, HepG2, HCC-S102, SK-Hep-1, and Alexander, suggesting the expression might be specific to CCA [43]. It has also been found that serum *LCN2* levels were significantly elevated in ICC patients compared to those with benign biliary tract disease [44]. In a separate study, no significant differences were found in sera between malignant and benign biliary patients, whereas elevated *LCN2* levels were detected in bile collected from the ICC patients [45]. Moreover, the roles of LCN2 in ICC have been studied by knocking down *LCN2* with siRNA that resulted in a significant reduction in invasiveness, migration and pro-MMP-9 activity of ICC cells [46].

To further verify *LCN2* role in human ICC, we examined 80 human ICC specimen and 66% of human ICC specimen presented with high expression *LCN2*, adding the finding that knockdown of *LCN2* in SNU308 cells decreased cell growth and migration (Fig 4f), indicating the *LCN2* plays as an oncogene in human ICC. In addition, as application of 1α,25(OH)_2_D_3_ to treat human ICC SNU1079 cells, which express VDR (Fig. 4a), a dose dependent antiproliferation and downregulation of *LCN2* mRNA and protein expressions was observed (Fig.4b,c,&d). Of note, as most cancer cells respond to 10^−8^M 1α,25(OH)_2_D_3_ [17], 10^−10^M 1α,25(OH)_2_D_3_ significantly inhibited SNU1079 cell growth (Fig 4b), implicating the much more sensitivity of SNU1079 cells to 1α,25(OH)_2_D_3_ as compared to other cancer cells. Since 1α,25(OH)_2_D_3_ exerts its genomic function through binding with VDR, we next knocked down VDR in SNU1079 cells and the inhibition of *LCN2* expression by 1α,25(OH)_2_D_3_ was attenuated (Fig. 4e), indicating this effect of 1α,25(OH)_2_D_3_ is VDR-dependent, which is also supported by the finding that the SNU-VDRsi cells presented with higher *LCN2* expression as compared to that of SNU-COLsi cells since the added FBS in cell culture medium contained some amount of 1 α,25(OH)_2_D_3_ (Fig 4e). These finding further suggested that the *in vivo* downregulation of *LCN2* in this current animal model is the direct consequence of vitamin D supplementation.

Collectively, we concluded that *LCN2* is an oncogene in human ICC and its expression is repressed by 1α,25(OH)_2_D_3_ VDR-dependently. Thus, the downregulation of Lcn2 in rats ICC tissues is supposed to be a direct sequence by vitamin D supplementation, leading to the observed in vivo anti-ICC tumorgenesis. Our present results are consistent with previous studies suggesting that 1α,25(OH)_2_D_3_ and its analogs are able to inhibit ICC tumor growth *in vitro* and *in vivo* [47-49].

In summary, we demonstrated in this report that vitamin D in a form of dietary supplementation can prevent and suppress ICC tumorigenesis and progression with downregulation of *LCN2* in an animal model without inducing hypercalcemia. Furthermore, the results showing that the high expression of *LCN2* in human ICC specimen, the decreased proliferation and migration of SNU308 cells after *LCN2* knockdown, and the 1α,25(OH)_2_D_3_-induced antiproliferative effect and VDR-dependent downregulation of *LCN2* in SNU1079 cells, strongly suggest *LCN2* may be a new target against human CCA. Thus, based on our results, we concluded that maintaining adequate vitamin D status has the potential to be an inexpensive and effective approach against ICC. Further investigations to determine the optimal amounts of vitamin D supplementation required for cancer prevention and the application of vitamin D and its D analogs for the treatment of ICC are warranted.

## MATERIALS AND METHODS

### Induction of intrahepatic cholangiocarcinoma (ICC) in rats

Male Sprague-Dawley (SD) rats weighting 250 ± 14 g were obtained from BioLasco Taiwan Co., Ltd (Taipei, Taiwan). Animals were divided into three groups (N=7 per group) and fed with a diet supplemented with no vitamin D_3_ (cat. # 5A0E), 2 IU/g of vitamin D_3_ (cat.# 5A0G), or 6 IU/g of vitamin D_3_ (cat. # 5A0J) obtained from TestDiet (Richmond, IN, USA). Animals were housed in an animal room with a 12-hour incandescence light and dark cycle at an ambient temperature of 22°C with food and water available *ad libitum*, and avoid of any natural sunlight and fluorescence lighting during the course of study. The animal protocol was approved by the Experimental Animal Ethics Committee of Chang Gung Memorial Hospital (Approval: IACUC 2011081901) Beginning in week 8, drinking water was replaced with water containing 0.03% TAA.

### Measurements of body weight, serum calcium and serum 25-hydroxyvitamin D [25(OH)D]

Measurement of body weight and blood drawing for calcium and 25(OH)D analyses were taken upon the arrival of animals and in week 4, 6, 11, 17, 23, 26, and 31. Serum calcium and serum 25(OH)D were analyzed using kits obtained from Stanbio Laboratory (CALCIUM LIQUICOLOR^®^ (ARSENAZO) (#0155-225, Stanbio Laboratory, TX, USA)) and from DiaSorin (25-Hydroxyvitamin D ^125^I RIA kit (#68100E, DiaSorin, MN, USA)), respectively. The calcium kit has intra- and inter-assay coefficient of variation of 0.7 and 0.9%, respectively. The 25(OH)D assay kit has intra- and inter-assay *cv* of 8.2 and 10.5%, respectively. The limit of detection is 1.5 ng/ml for 25(OH)D and 1mg/dl for calcium.

### Detection of TAA-induced ICC tumors by position emission tomography (PET) and histopathological evaluation of the liver

The detection of ICC tumors was accomplished by injecting 2-deoxy-2-[F-18]fluoro-D-glucose (FDG) 90 minutes prior to PET scan which was performed on an Inveon™ system (Siemens Medical Solutions, Inc. USA) located in the Molecular Image Center of Chang Gung Memorial Hospital as described previously [29]. Scan was carried out once a week for six consecutive weeks beginning in the 20^th^ week after the addition of TAA into the drinking water. Quantification of ^18^F-FDG uptakes in the biggest liver tumor and normal liver tissue was performed by calculating the standardized uptake value (SUV) as previously described [29].

### Microarray analysis of ICC

Three TAA induced cholangiocarcinoma samples from each group of rats fed with a diet supplemented with no vitamin D, 2 IU/g vitamin D_3_ or 6 IU/g vitamin D_3_ were used for microarray analysis. Rat OneArray® v1 (Phalanx Biotech Group, Hsinchu, Taiwan) was chosen for its reproducibility in examining the quantitative and qualitative expression of most genes in the rat genome. Following a quantitative scan of a chip, the images were transformed to text files containing intensity information by Phalanx^®^ and the microarray data were analyzed by using the GeneSpring^®^ GX 7.3.1 Software (Agilent Technologies, Santa Clara, CA, USA). The further descriptions were shown in supplementary materials.

### Immunohistochemical staining (IHC) of rat and human ICC tissues for *LCN2*

Human ICC tumors were obtained from patients admitted to the Chang Gung Memorial Hospital. The protocol was approved by the IRB of the Chang Gung Memorial Hospital (Approval: IRB 99-2886B). The detailed procedures were dercribed in the supplemental material section.

### Cell culture

SNU1079 cells and SNU308 cells were obtained from Korean Cell Line Bank (KCLB: 28 Yongon-dong, Chongno-gu, Seoul 110-744, Korea). Cells were grown on RPMI 1640 medium supplemented with 10% FBS and 1% antibiotic-antimycotic agents. Culture medium was changed 3 times per week.

### Knockdown of VDR and real-time qPCR of *LCN2* expression in Human ICC SNU1079 cells

SNU1079 cells were transduced with control non-target shRNA lentiviral particles (SI-SHC002V, Sigma, MO, USA) or vitamin D receptor (VDR) shRNA lentiviral particles (SI-NM_000376.1-578s1, Sigma). Four days after transduction, the cells (SNU-COLsi and SNU-VDRsi) were treated with 10-7 M of 1α,25(OH)_2_D_3_ or control vehicle for 24 hours. Total RNA was isolated using the Trizol reagent, and cDNA was synthesized using the Superscript III preamplification system (Invitrogen). FAM dye-labeled TaqMan MGB probes and PCR primers for human *LCN2* (HS00194353-m1), CYP24A1 (HS00167999-m1), and VDR (HS01045844-m1) were purchased from Applied Biosystems. For the internal positive control, GAPDH (HS99999905-m1) was used with a FAM reporter dye-labeled TaqMan MGB probe. Mean cycle threshold (C*_t_*) values for *LCN2*, CYP24A1, and VDR were normalized against the GAPDH control probe to calculate ΔC*_t_* values using StepOne software v2.0 (Applied Biosystems).

### Knockdown of *LCN2* in human ICC SNU308 cells

SNU308 cells were transducted with control small hairpin RNA lentiviral particles (Sc-10808-V, Santa Cruz Biotechnology) or NGAL small hairpin RNA lentiviral particles (Sc-43969-V, Santa Cruz Biotechnology) according to the manufacturer's instructions. Two days after transduction, the cells (SNU-COLsi and SNU-NGALsi) were selected by incubation with 10 μg/ml puromycin dihydrochloride for another 3 generations.

### Real-time qPCR (RT-qPCR) and western blot analysis of rat tumors

Total RNA extraction and RT-qPCR (Lcn2 primer: All-in-One™ qPCR Primer, #RQP052324, GeneCopoeia, Rockville, MD, U.S.A.) were performed as described in the previous section. The procedures for protein extraction, blocking, and detection for western blot were performed as the manufacture guideline. The primary antibodies used in this study were *LCN2* polyclonal antibody (#PAB9543, 1:1000, Abnova Corporation, Taipei, Taiwan). The secondary antibodies were Goat Anti-Rabbit IgG (H+L) HRP Antibody (#3053-1, 1:10000, Epitomics, CA, USA).

### Cell proliferation assay by WST-1 kit

SNU1079 cells were plated at about 1,000 cells per cm^2^ in a Costar® 48 Well Clear TC-Treated Multiple Well Plates (#3548, Corning Incorporated, NY, USA). The cells were treated with ethanol vehicle (control group) or 1α,25(OH)_2_D_3_ at the indicated concentrations. The viable cells were measured by Cell Proliferation Reagent WST-1 (#11 644 807 001, Roche Diagnostrics, Mannheim, Germany).

### Trans-well filter migration assay

SNU-COLsi and SNU-NGALsi cells were seeded on each trans-well filter with 8.0-μm pores (Costar, Cambridge, MA, USA). The upper chamber was filled with 250 μl serum-free DMEM and the lower chamber was filled with 600 μl DMEM with 10% FBS. These cells were allowed to migrate at 37°C in an atmosphere of 95% air-5% CO_2_ for 16 h. Cells that migrated through the pores were stained with Liu's stain and washed with 1xPBS twice. Then the cells on the lower surface of the filter were counted under four random high-power microscopic fields (HPF;100X) per filter, and the mean number of cells that migrated through the filter was calculated for each condition. The experiments were performed in triplicates.

### Statistical analysis

OneWay ANOVA with Post Hoc test was used for the statistical analysis of SUV for ^18^F-FDG uptakes among the three treatment groups during PET scanning. For human *LCN2* IHC staining comparison, the differences among the three groups were analyzed by Kruskal-Wallis test, whereas the difference between each two groups were analyzed by Dunn's Multiple Comparison Test. The Student t-test was used for the statistic analyses of serum calcium, serum 25(OH)D, body weight and in vitro experiments. P-value<0.05 was considered as a significant difference. The program of Excel 2007 or SPSS statistical software for Windows (SPSS version 10.0, Chicago, IL, USA) were employed to conduct statistical analysis.

## SUPPLEMENTARY MATERIAL AND FIGURES


