# Supplementary material for: Chemopreventive and chemotherapeutic effects of dietary supplementation of vitamin D on cholangiocarcinoma in a Chemical-Induced animal model
Source: Oncotarget. 2014 May 22;5(11):3849–61. doi: 10.18632/oncotarget.2000 (PMC4116525; doi:10.18632/oncotarget.2000)
Supplement: Supplementary file 1 [file oncotarget-05-3849-s001.pdf]

Chemopreventive and Chemotherapeutic Effect of Dietary Supplementation of Vitamin D on Cholangiocarcinoma in a Chemical-Induced Animal Model

Supplementary Material

Microarray analysis of ICC

Three TAA induced cholangiocarcinoma samples from each group of rats fed with a diet supplemented with no vitamin D, 2 IU/g vitamin D_3 or 6 IU/g vitamin D_3 were used for microarray analysis. Rat OneArray® v1 (Phalanx Biotech Group), which covers 24,358 well-substantiated rat transcripts, was chosen for its reproducibility in examining the quantitative and qualitative expression of most genes in the rat genome. Fluorescent aRNA targets were prepared from 2.5 μg total RNA samples using OneArray® Amino AllylaRNA Amplification Kit (Phalanx Biotech Group, Taiwan) and Cy5 dyes (Amersham Pharmacia, Piscataway, NJ, USA). Fluorescent targets were hybridized to the Rat Whole Genome OneArray® with Phalanx hybridization buffer using Phalanx Hybridization System. After 16 hrs hybridization at 50 °C, non-specific binding targets were washed away by three different washing steps (Wash Ⅰ 42 °C 5 mins；Wash Ⅱ 42 °C, 5 mins, 25°C 5 mins; Wash Ⅲ rinse 20 times), and the slides were dried by centrifugation and scanned by an Axon 4000B scanner (Molecular Devices, Sunnyvale, CA, USA). The Cy5 fluorescent intensities of each spot were analyzed by GenePix 4.1 software (Molecular

Devices).

The signal intensity of each spot was loaded into Rosetta Resolver System® (Rosetta Biosoftware) to process data analysis. The error model of Rosetta Resolver System® could remove both systematic and random errors form the data. We filtered out spots that the flag is less than 0. Spots that passed the criteria were normalized by 50% media scaling normalization method. The technical repeat data were tested by Pearson correlation coefficient calculation to check the reproducibility (R value > 0.95). Normalized spot intensities were transformed to gene expression log2 ratios between the control and treatment groups. The spots with log2 ratio ≥ 1 or log2 ratio ≤ -1 and P-value < 0.05 were tested for further analysis.

Data analysis, clustering algorithm, and gene ontology analysis

Following a quantitative scan of a chip, the images were transformed to text files containing intensity information by Phalanx®. The microarray data were analyzed using the GeneSpring® GX 7.3.1 Software (Agilent Technologies, Santa Clara, CA, USA). A statistical comparison of gene expression between the matched normal/tumor specimens was performed using the Kruskal-Wallis test ($p < 0.01$), and this approach used false-discovery rate (FDR) to account for simultaneous testing on thousands of genes (multiple testing correction). The significant difference in

expression was set as 1.5-fold changes. To evaluate gene expression patterns, hierarchical clustering was carried out by the Pearson's correlation metric and average linkage. Differential express genes were subjected to principal component analysis (PCA) to determine patterns in the variability of expression profiles. Signaling pathway analysis was performed using the Database for Annotation, Visualization and Integrated Discovery (DAVID) v6.7 (http://david.abcc.ncifcrf.gov/) to distinguish differential distribution(28).

Hierarchical clustering resulted in the identification of 1,633 transcripts, which were highly affected by vitamin D supplementation in the TAA induced rat ICC tumors, as illustrated in a diagram format (Fig. 2a). Furthermore, multivariable analysis by PCA method identified very different array profile patterns among the control, +2D and +6D groups, while similar array profiles were obtained within the group (Fig. 2b).

The genes with significant expression changes were further examined by bioinformatics' analysis using DAVID. Pathway information is one important area for understanding the functionality of genes and proteins. Interestingly, the top 18 signal pathways (10 up and 8 down regulated-related) showed strong association with retinol, fatty acid and drug metabolism (Table 1a). By using more stringent criteria, we were able to narrow down to 21 and 16 transcripts, respectively, which exhibit 1.5-fold up and down regulated expression (Table 1b). Of these 37 differentially expressed

genes, the expression pattern of *Lcn2* (lipocalin 2) was found to be the most evident (Fig. S-1).

Immunohistochemical staining (IHC) of rat and human ICC tissues for LCN2

Human ICC tumors were obtained from patients admitted to the Chang Gung Memorial Hospital. The protocol was approved by the IRB of the Chang Gung Memorial Hospital (Approval: IRB 99-2886B). For the immunostaining of rat Lcn2 or human LCN2/NGAL, slides were incubated with primary polyclon antibody against Lcn2 (#AB2267, 1:200, Millipore Corporation, MA, USA) or monoclonalHuman Lipocalin - 2/NGAL Antibody (#MAB1757,1:50, R&D Systems, MN, U.S.A) overnight at 4°C. The slides were then washed three times, 5 minutes per wash, in TBST before visualization with Universal LSAB™2 Kit/HRP, Rabbit/Mouse (#K0675, DakoCytomation Inc., CA, USA). The expression of LCN2/NGAL in human tumor was categorized into weak (1+), intermediate (2+), and strong (3+) positive stain (Figure S-2 in the Supplemental Materials Section).

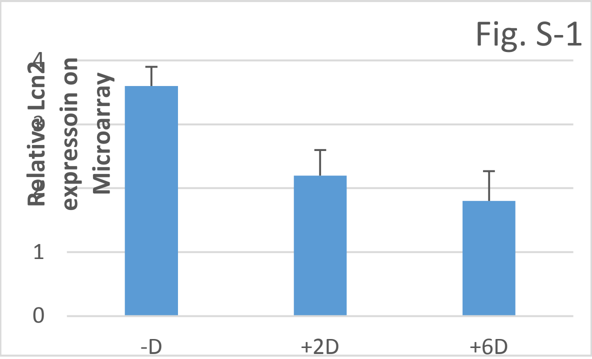

Figure S-1: Gene expression profile of Lcn2 in TAA induced-ICC tumors obtained from –D, +2D and +6D rats by microarray analysis.

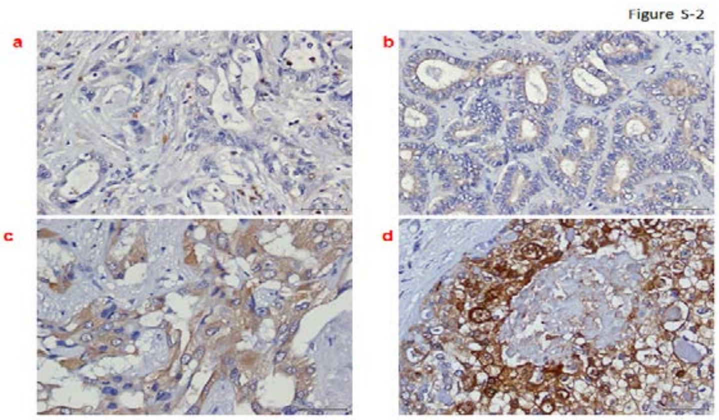

Figure S-2: Expression analysis of human LCN2/NGAL by immunohistochemistry. LCN2/NGAL was mainly localized in the cytoplasm of cholangiocarcinoma cells (x400). Brown color indicates positive staining with LCN2/NGAL. (a) Negative staining in non-cancerous tissue, (b) weak staining, 1+ positive, (c) intermediate staining, 2+ positive, (d) strong staining, 3+ positive.

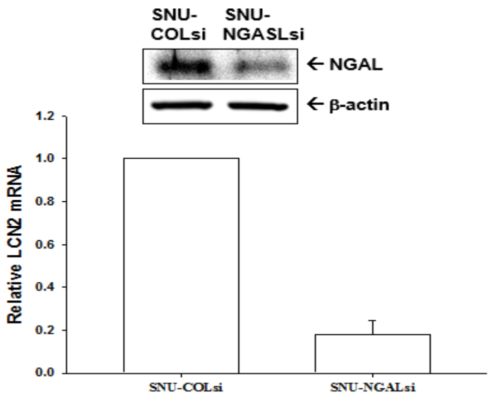

Figure S-3: Knockdown of LCN2 in SNU308 cells

Upper panel :A western blot showing NGAL expression in SNU-COLsi cells (mock knockdown SNU308 cells) and SNU-NGALsi cells (LCN2 knockdown SNU308 cells) with β-actin serving as internal control

Lower panel : Quantitative analysis of LCN2 mRNA level in SNU-COLsi cells and SNU-NGALsi cells with the formal set as 1.
